# A prospective cohort study on effects of mandibular setback with or without maxillary advancement for skeletal class III malocclusion on sleep-related respiratory parameters

**DOI:** 10.1007/s11325-025-03347-7

**Published:** 2025-04-30

**Authors:** Ratanaporn Patharakorn, Nuntinee Nanthavanich Saengfai, Chaiyapol Chaweewannakorn, Supatchai Boonpratham, Yodhathai Satravaha, Supakit Peanchitlertkajorn

**Affiliations:** Department of Orthodontics, Faculty of Dentistry, Mahidol University, Bangkok, Thailand

**Keywords:** Skeletal class III malocclusion, Orthognathic surgery, Obstructive sleep apnea, Sleep-related respiratory parameters

## Abstract

**Purpose:**

This study aimed to investigate changes in sleep-related respiratory parameters before and after orthognathic surgery in patients with skeletal class III malocclusion.

**Methods:**

Adults with skeletal class III malocclusion and treated with isolated mandibular setback or bimaxillary surgery (maxillary advancement and mandibular setback) were recruited. Sleep-related respiratory parameters were obtained with type III sleep study. Epworth Sleepiness Scale (ESS) was also recorded. The pre- and post-operative (6 months) data were compared. Correlations between these changes and pre-operative characteristics were analyzed. Subjects were categorized into three groups based on changes in the respiratory event index (REI) and 3% oxygen desaturation index: Δ ≤ -2.5, -2.5 < Δ < 2.5, and Δ ≥ 2.5. Amounts of surgical movement and pre-surgical parameters were compared among the 3 groups.

**Results:**

Thirty patients with an average age of 25.4 ± 5.0 years were recruited. Eleven patients underwent isolated mandibular setbacks while nineteen received bimaxillary surgery. Pre- and post-operative sleep-related respiratory parameters were not significantly different in the total samples, and when analyzed separately according to surgical procedures. Pre-operative ESS were correlated with the changes in REI (*p* = 0.01), average blood oxygen levels (*p* = 0.01), and snoring percentage (*p* = 0.04). Additionally, this study found that patients with a significant decrease in REI (ΔREI ≤ -2.5) after surgery had significantly higher pre-operative REI (6.2 events/hour) compared to those with minor REI changes (2.6 events/hour).

**Conclusion:**

There was no significant change in sleep-related respiratory parameters following mandibular setbacks with or without maxillary advancement in this study.

**Trail registered:**

This study was retrospectively registered and approved on February 11, 2025, under registration number TCTR20250211002.

## Introduction

Skeletal class III malocclusion is one of the most challenging problems in orthodontics. The prevalence varied across different races and ethnic groups, and was found to be highest in Southeast Asian population [1]. Among available treatment options, orthognathic surgery remains a viable approach. Previously, isolated mandibular setback surgery (MS) was the standard orthognathic procedure. However, the number of bimaxillary surgery (BS), a combination of maxillary advancement and mandibular setback, increased and became the most common procedure for correcting skeletal class III malocclusion. Previous research reported a decrease in pharyngeal airway space (PAS) observed on cephalogram in skeletal class III patients following both types of surgery correction [2]. Long-term study found that PAS constriction remained significant in patients treated with MS even two years after, potentially predisposing them to the development of obstructive sleep apnea (OSA) [3]. In contrast, patients undergoing BS showed no significant PAS reduction after this period [3]. Additionally, a volumetric study demonstrated that BS leads to a smaller reduction in airway volume compared to MS [4]. However, it is crucial to recognize that neither 2-dimensional (2D) nor 3-dimensional (3D) imaging accurately represents the dynamic function of the upper airway during sleep and cannot be used as diagnostic tools for OSA.

OSA is a condition characterized by a temporary cessation or significant reduction in airflow exchange during sleep [5, 6]. This leads to a decreased oxygen supply to the brain, heart, and other vital organs, and potentially causes serious health problems such as systemic hypertension, cognitive impairment, cardiac arrhythmias, and stroke [5, 6]. The prevalence of OSA varies among different racial and ethnic groups. Despite lower obesity rates, Asians have been found to experience more severe OSA than Caucasians even after adjusting for age and body mass index (BMI) [7]. This discrepancy may be attributed to differences in craniofacial anatomy such as a retrognathic mandible, which could narrow the upper airway and increase the likelihood of OSA. A meta-analysis identified mandibular body length as the craniofacial measure most strongly associated with an increased risk of OSA [8]. Other major risk factors for OSA include age, body weight, and gender, with higher prevalence observed among individuals of advanced age, those with obesity, and males [9].

The gold standard for diagnosing OSA is polysomnography (PSG). The American Academy of Sleep Medicine (AASM) categorizes sleep studies into four types based on the availability of sleep technicians, location, and number of parameters recorded [5]. While Type I provides more accurate and reliable results, it comes with higher costs, inconvenience, and limited accessibility [5]. Alternatively, Type III sleep study, also known as a home sleep test (HST), generally offers a more cost-effective and accessible alternative [10]. A systematic review and meta-analysis comparing HST to type I PSG demonstrated that HST has a comparable diagnostic performance, with high sensitivity and specificity [11]. The apnea-hypopnea index (AHI) is currently the primary metric for diagnosing OSA and assessing its severity in PSG [6]. Due to absence of electroencephalography (EEG) to detect sleep stages and estimate total sleep time, many HST calculates respiratory event using total analyzed time instead and reports respiratory event index (REI) instead of AHI [5].

A recent systematic review and meta-analysis of 476 patients demonstrated that sleep-related parameters including AHI, respiratory disturbance index (RDI), minimum blood oxygen levels (min SpO_2_), average blood oxygen levels (average SpO_2_), and the 3% oxygen desaturation index (3% ODI), were not significantly different after mandibular setback with or without maxillary advancement for skeletal class III correction surgery. The study also found that the two different orthognathic procedures did not significantly impact post-operative AHI, average SpO_2_, and 3% ODI. However, 5.8% of patients developed post-operative OSA, especially those who underwent large amounts of mandibular setback [12]. In addition, previous retrospective studies showed non-significant changes in PSG parameters following mandibular setback, regardless of maxillary advancement [13, 14]. These types of studies, however, are often presented potential selection biases. Although several prospective studies have been performed, they examined only pre- and post-operative differences in either MS [15, 16] and BS [17–20] separately. To the best of our knowledge, only two studies investigated both interventions by comparing them but reported contrasting results [21, 22]. Uesugi et al. found non-significant changes in post-operative AHI for both groups [21], whereas On et al. showed a significant increase in RDI following MS [22]. Furthermore, none of previous studies performed sample size calculation [16, 17, 21, 22]. This raised concerns regarding the adequacy of statistical power and validity of these results. Therefore, the primary objective of this study was to perform a prospective cohort study to evaluate changes in sleep-related respiratory parameters before and after MS versus BS in patients with skeletal class III malocclusion. In addition, we examined potential correlations between changes in the parameters and pre-operative variables. Finally, we compared pre-operative variables and the amount of surgical movement across groups of patients exhibiting different degrees of changes in REI and 3% ODI.

## Materials and methods

This prospective cohort study was conducted at the Dental Hospital, Faculty of Dentistry, Mahidol University. Ethical approval for the study was granted by the Institutional Review Board of the Faculty of Dentistry, Mahidol University (COA.No.MU-DT/PY-IRB 2021/004.0801). Informed consent was obtained from all subjects prior to their participation. Adult patients with skeletal class III (Wits appraisal less than − 5 mm) [23] with a plan to undergo orthognathic surgery, were recruited. Exclusion criteria were patients with the following conditions: craniofacial deformities, cardiopulmonary diseases, hypothyroidism, polycystic ovary syndrome (PCOS), pregnancy, history of orthognathic surgery, history of ENT surgery (including uvulopalatopharyngoplasty, tonsillectomy, and adenoidectomy), and BMI > 35 kg/m^2^. The decision to perform MS or BS was determined primarily by the amount of jaw discrepancy (BS for larger discrepancy).

Demographic data, Epworth Sleepiness Scale (ESS), and sleep-related respiratory parameters were collected at two time points: immediately before surgery (T0) and at six months post-operatively (T1). The sleep test was performed using Nox-T3 system (Nox Medical Inc. Reykjavik, Iceland), a type III sleep study approved by the FDA [10]. The following parameters were evaluated: REI, 3% ODI, average SpO_2_, min SpO_2_, and snoring percentage. In addition, the amounts of surgical movement were acquired from the virtual surgical plans generated by Dolphin Imaging 11.95 Premium^®^ (Dolphin Imaging and Management Solutions, Chatsworth, CA, USA). The distances were measured in the antero-posterior (A-P) direction, utilizing the Frankfurt horizontal plane as a reference line. The distances measured were maxillary movement (A), upper incisor movement (UI), mandibular movement (B), and lower incisor movement (LI).

The sample size calculation was based on Uesugi et al.‘s study [21]. The calculation resulted in a minimum of 11 subjects for each surgical group with 80% power to identify a REI difference (*p*-value < 0.05) of 2.5 events/hour.

Data distribution was examined using the Shapiro-Wilk test. Pre- and post-operative parameters were then compared in 3 groups: MS, BS, and the total samples (MS combining with BS). The Wilcoxon signed-rank test was performed when the data did not follow a normal distribution. These included the REI, 3% ODI, and min SpO_2_ in the total samples and the BS group, as well as BMI and waist circumference in the MS group, and snoring percentage in all three groups. Parameters not mentioned above followed a normal distribution and were analyzed using a paired t-test.

Pearson’s correlation coefficients were performed to examine correlations between age, pre-operative BMI, pre-operative ESS, and differences between the pre- and post-operative sleep-related respiratory parameters. The differences between genders were evaluated by comparing the changes between pre- and post-operative sleep-related respiratory parameters. Average SpO_2_ and min SpO_2_ differences between T0 and T1 were analyzed using independent t-tests. Differences in other PSG parameters (REI, 3% ODI, snoring percentage) were analyzed using Mann-Whitney U test.

Additionally, the mean amount of surgical movement and other pre-operative variables were compared among 3 groups of patients categorized by changes in REI and 3% ODI: clinically significant increase (Δ ≥ 2.5 events/hour), clinically significant decrease (Δ ≤ -2.5 events/hour) and clinically insignificant change (-2.5 < Δ < 2.5 events/hour). For group comparisons based on the REI differences, the following variables: mandibular movement, neck circumference, waist circumference, average SpO_2_, min SpO_2_, and ESS, were analyzed using one-way ANOVA. The Bonferroni analysis was used for pairwise comparisons. Other variables based on REI and 3% ODI changes were analyzed using the Kruskal-Wallis test. All statistical analyses were conducted using Stata (16.1, StataCorp LLC, College Station, TX). The significance level was set at *p*-value of 0.05.

## Results

Thirty patients (20 males and 10 females) participated in this study. The average age and BMI were 25.4 ± 5.0 years, and 21.9 ± 2.8 kg/m^2^, respectively. The pre-operative ESS was 8.3 ± 4.3, indicating that participants, on average, exhibited a normal level of daytime sleepiness [24]. The pre-operative sleep-related respiratory data across REI (< 5, and ≥ 5 events/hour) and gender subgroups are presented in Table 1. Eleven patients underwent MS, while nineteen patients underwent BS. The mean distance of mandibular setback in the MS group was 5.8 ± 3.0 mm. The maxilla was advanced 3.2 ± 1.1 mm with the mandible being setback 7.7 ± 4.4 mm on average in the BS group. Across the entire samples, the mean mandibular setback was 7.0 ± 4.0 mm. In addition, 25% of participants in this study were found to have their REI increased above 5 events/hour following MS and BS. Among subjects with a pre-operative REI ≥ 5 events/hour, none exhibited a change in OSA severity following surgery. The median (IQR) follow-up period was 221.5 (194.8–288.5) days.

**Table 1:** Comparison of pre-operative sleep-related respiratory parameters across REI and gender subgroups. Data are presented as mean (minimum and maximum range)

Pre-operative REI	< 5 events/hour		≥ 5 events/hour	
Gender	Male (*n* = 11)	Female (*n* = 9)	Male (*n* = 9)	Female (*n* = 1)
REI (events/hour)	2.3 (0.0–4.5)	2.2 (0.6–4.7)	8.4 (5.4–15.8)	5.9
3% ODI (events/hour)	2.7 (0.3–6.9)	1.5 (0.3–2.8)	7.4 (3.3–12.3)	3.3
Average SpO_2_ (%)	96.4 (94.9–97.5)	96.4 (94.6–97.9)	95.5 (94.4–96.5)	96.5
Min SpO_2_ (%)	87.6 (81.0–95.0)	85.8 (79.0–94.0)	82.2 (69.0–92.0)	93
Snoring percentage (%)	1.0 (0.0–4.3)	0.6 (0.0–4.7)	5.4 (0.0–24.9)	0
ESS	10.2 (6.0–19.0)	7.3 (2.0–14.0)	8.0 (2.0–15.0)	0
BMI (kg/m^2^)	22.6 (18.1–29.1)	21.0 (18.3–27.6)	22.3 (17.3–24.7)	19
Age (years)	25.7 (21.0–41.0)	24.3 (20.0–38.0)	26.2 (21.0–32.0)	24

There was no significant difference observed between pre- and post-operative PSG parameters as well as ESS in the total samples, and the 2 different surgical procedure groups (MS and BS). Significant reduction in BMI, neck circumference, and waist circumference were found in the total samples and the BS group. Table 2 provides detailed comparisons of the pre- (T0) and post-operative (T1) parameters.

**Table 2:** Comparison of pre- and post-operative sleep-related respiratory parameters. Data are presented as mean (standard deviation) for normally distributed variables and as median (minimum and maximum range) for variables that do not follow a normal distribution

Parameters	Total samples (*n* = 30)			Mandibular setback surgery (*n* = 11)			Bimaxillary surgery (*n* = 19)		
	Pre-operative	Post-operative	*p*-value	Pre-operative	Post-operative	*p*-value	Pre-operative	Post-operative	*p*-value
REI (events/hour)	4.0 (0.0–15.8)	3.9 (0.0–20.1)	0.61	3.0 (2.2)	4.1 (2.9)	0.19	4.2 (0.0–15.8)	3.6 (0.0–20.1)	0.86
3% ODI (events/hour)	2.9 (0.3–12.3)	2.9 (0.4–16.3)	0.28	2.8 (2.2)	3.1 (2.2)	0.63	3.0 (0.3–12.3)	3.6 (0.5–16.3)	0.28
Average SpO_2_ (%)	96.1 (1.0)	96.4 (0.8)	0.15	96.3 (1.1)	96.5 (0.8)	0.57	96.1 (0.9)	96.4 (0.7)	0.10
Min SpO_2_ (%)	84.0 (69.0–95.0)	84.0 (52.0–94.0)	0.34	87.0 (6.7)	86.2 (4.4)	0.68	84.0 (69.0–95.0)	83.0 (52.0–92.0)	0.35
Snoring percentage (%)	0.1 (0.0–24.9)	0.2 (0.0–35.4)	0.28	0.0 (0.0–24.9)	1.0 (0.0–35.4)	0.07	0.3 (0.0–9.5)	0.2 (0.0–18.8)	0.58
ESS	8.3 (4.3)	8.5 (4.4)	0.83	9.6 (5.9)	9.3 (4.8)	0.86	7.6 (2.8)	8.0 (5.9)	0.49
BMI (kg/m^2^)	21.9 (2.8)	21.0 (2.1)	< 0.01*	21.9 (19.0–29.1)	21.5 (18.6–25.3)	0.25	21.5 (2.6)	20.6 (2.0)	< 0.01*
Neck circumference (cm)	34.0 (3.4)	33.4 (3.1)	< 0.01*	34.4 (3.3)	33.6 (3.3)	0.07	33.8 (3.5)	33.2 (3.1)	0.03*
Waist circumference (cm)	77.0 (8.8)	74.0 (6.8)	< 0.01*	79.0 (61.0–93.0)	76.0 (61.5–81.5)	0.25	77.0 (9.1)	73.6 (7.3)	< 0.01*

*p <.05

Pre-operative ESS exhibited a moderate positive correlation with REI difference (Pearson’s Coefficient = 0.44, *p*-value = 0.01), a moderate negative correlation with average SpO_2_ difference (Pearson’s Coefficient = -0.46, *p*-value = 0.01), and a weak positive correlation with snoring percentage difference (Pearson’s Coefficient = 0.36, *p*-value = 0.04). No significant correlation was observed for the other variables analyzed. Further details are presented in Table 3. Additionally, when comparing the changes in sleep-related respiratory parameters between sexes, this study found no significant differences between male and female subjects (Table 4).

**Table 3:** Correlations between changes in sleep-related respiratory parameters and pre-operative variables

Parameters	Pearson’s coefficient (*p*-value)		
	Age	Pre-operative BMI	Pre-operative ESS
REI difference (events/hour)	0.10 (0.60)	0.12 (0.54)	0.44 (0.01) *
3% ODI difference (events/hour)	-0.02 (0.91)	0.05 (0.78)	0.16 (0.40)
Average SpO_2_ difference (%)	-0.12 (0.53)	-0.17 (0.36)	-0.46 (0.01) *
Min SpO_2_ difference (%)	0.20 (0.29)	0.05 (0.79)	-0.13 (0.48)
Snoring percentage difference (%)	0.12 (0.53)	0.06 (0.74)	0.36 (0.04) *

*p <.05

**Table 4:** Comparison of changes in sleep-related respiratory parameters between sexes. Data are presented as mean (standard deviation) for normally distributed variables and as median (minimum and maximum range) for variables that deviate from normal distribution

Parameters	Male (*n* = 20)	Female (*n* = 10)	*p*-value
REI difference (events/hour)	1.1 (-7.6–5.5)	-1.1 (-4.2–0.9)	0.07
3% ODI difference (events/hour)	0.7 (-2.6–13.5)	0.4 (-2.4–3.7)	0.66
Average SpO_2_ difference (%)	0.2 (0.8)	0.5 (1.4)	0.56
Min SpO_2_ difference (%)	-3.1 (10.7)	-0.2 (7.1)	0.20
Snoring percentage difference (%)	0.1 (-3.7–10.5)	0.0 (-4.7–4.0)	0.60

When subjects were categorized into three groups based on their pre- and post-operative REI differences (clinically significant increase, clinically significant decrease, and clinically insignificant change), a statistically significant difference was observed only in pre-operative REI. Pairwise comparisons revealed a significant difference between the clinically significant decrease group (ΔREI ≤ -2.5 events/hour) and the clinically insignificant change group (-2.5 < ΔREI < 2.5 events/hour). Details are shown in Table 5.

**Table 5:** Comparison of the amount of surgical movement and pre-operative variables among 3 groups of patients with different degrees of REI changes following the surgery. Data are reported as mean (standard deviation) for variables with a normal distribution and as median (minimum and maximum range) for variables that deviate from normality

Parameters	REI changes (events/hour)			
	Clinically significant decrease group Δ ≤ -2.5 (*n* = 6)	Clinically insignificant change group -2.5 < Δ < 2.5 (*n* = 16)	Clinically significant increase group Δ ≥ 2.5 (*n* = 8)	*p*-value
Maxillary advancement distance (mm)	3.0 (0.0–3.5)	1.8 (0.0–6.5)	1.8 (0.0–3.7)	0.84
Mandibular setback distance (mm)	6.7 (4.1)	8.0 (4.3)	5.3 (3.2)	0.30
Pre-operative BMI (kg/m^2^)	23.5 (17.3–25.0)	21.0 (18.1–27.6)	21.7 (20.2–29.1)	0.57
Pre-operative Neck circumference (cm)	34.8 (4.2)	32.8 (3.3)	35.8 (2.1)	0.09
Pre-operative Waist circumference (cm)	79.0 (11.9)	74.1 (7.3)	81.4 (7.6)	0.13
Pre-operative REI (events/hour)	6.2 (4.2–12.4) *	2.6 (0.0–6.9) *	2.1 (0.0–15.8)	0.04 *
Pre-operative 3% ODI (events/hour)	4.4 (2.8–11.4)	2.3 (0.3–8.5)	3.3 (0.3–12.3)	0.15
Pre-operative Average SpO_2_ (%)	95.5 (0.6)	96.3 (1.1)	96.4 (0.8)	0.14
Pre-operative Min SpO_2_ (%)	84.0 (5.0)	86.5 (6.0)	85.1 (8.7)	0.72
Pre-operative Snoring percentage (%)	0.2 (0.1–7.9)	0.0 (0.0–4.7)	0.2 (0.0–24.9)	0.13
Pre-operative ESS	7.0 (1.8)	7.3 (4.4)	11.4 (4.2)	0.05

*p <.05

Comparisons among the three groups based on 3% ODI changes before and after surgery revealed no significant difference in all variables. See Table 6 for additional details.

**Table 6:** Comparison of the amount of surgical movement and pre-operative variables among 3 groups of patients with different degrees of 3% ODI changes following the surgery. Data are reported as median (minimum and maximum range)

Parameters	3% ODI difference (events/hour)			
	Clinically significant decrease group Δ ≤ -2.5 (*n* = 1)	Clinically insignificant change group -2.5 < Δ < 2.5 (*n* = 24)	Clinically significant increase group Δ ≥ 2.5 (*n* = 5)	*p*-value
Maxillary advancement distance (mm)	0.0	2.5 (0.0–6.5)	1.9 (0.0–3.7)	0.53
Mandibular setback distance (mm)	1.0	6.8 (0.7–16.5)	7.5 (0.4–8.5)	0.31
Pre-operative BMI (kg/m^2^)	22.8	21.3 (17.3–29.1)	21.8 (20.2–27.6)	0.66
Pre-operative Neck circumference (cm)	39.0	34.3 (28.0–39.0)	34.0 (31.0–37.5)	0.27
Pre-operative Waist circumference (cm)	84.0	78.50 (61.0–93.0)	81.0 (72.0–85.5)	0.36
Pre-operative REI (events/hour)	5.4	3.3 (0.0–12.4)	5.6 (0.7–15.8)	0.62
Pre-operative 3% ODI (events/hour)	5.4	2.8 (0.3–11.4)	4.3 (0.3–12.3)	0.48
Pre-operative Average SpO_2_ (%)	95.3	96.2 (94.4–97.6)	96.5 (95.1–97.9)	0.39
Pre-operative Min SpO_2_ (%)	77.0	84.0 (78.0–95.0)	89.0 (69.0–94.0)	0.29
Pre-operative Snoring percentage (%)	2.8	0.1 (0.0–7.9)	0.3 (0.0–24.9)	0.28
Pre-operative ESS	6.0	7.0 (0.0–19.0)	11.0 (8.0–15.0)	0.07

## Discussion

This prospective cohort study investigated the effects of both MS and BS on sleep-related respiratory parameters in a group of Southeast Asian subjects. Our findings revealed non-significant changes in these parameters following the surgery, both when considering the entire samples together and when analyzing MS and BS groups separately. These findings are consistent with results from previous research [18, 20, 21], suggesting that the observed changes may fall within the range of physiological adaptation. However, two studies reported contrasting results, demonstrating significant increases in sleep-related respiratory parameters post-surgery [19, 22]. Yang et al. reported a significant rise in the AHI at six months after the surgery [19]. On et al. also observed a significant increase in the RDI three months following the surgery [22]. Notably, both studies involved limited maxillary advancement but substantial mandibular setback (On et al.: mean maxillary advancement = 1.57 mm, mean mandibular setback = 9.55 mm; Yang et al.: mean maxillary advancement = 0.65 mm, mean mandibular setback = 11.08 mm) [19, 22]. In addition, significant reductions in BMI, neck circumference, and waist circumference were observed post-surgically, especially in the BS group. Previous study suggested that changes in physical appearance, sensory perceptions, eating habits, oral function, invasiveness of the surgery, and the recovery process, could contribute to the observed weight reduction [25]. As excessive body weight is a risk factor for OSA [9], these significant reductions may have contributed to the non-significant changes observed in sleep-related respiratory parameters, potentially lowering the risk of OSA in our study population.

Additionally, this study found that pre-operative ESS was positively correlated with the changes in REI and snoring percentage, and negatively correlated with average SpO_2_. This suggested that patients with higher pre-operative daytime sleepiness could experience an increase in sleep-related respiratory disturbance and snoring, and a decrease in their average blood oxygen levels during sleep following the surgery. To our knowledge, this is the first study to identify these correlations. Therefore, we suggest administering the ESS to all skeletal class III patients undergoing orthognathic surgery and considering PSG for those with elevated ESS scores. This could help with identifying individuals at higher risk for developing post-operative OSA. Additionally, this observation may represent the first report indicating no significant differences between sexes regarding changes in PSG parameters following class III surgical correction. Previous research primarily examined the relationship between gender and OSA in a cross-sectional manner, highlighting the well-known higher prevalence of OSA in males, with a reported ratio of 2:1 [26]. This disparity has contributed to the under-recognition of OSA in women by healthcare providers. The lower prevalence of OSA in females was attributed to differences in symptom reporting, with women more frequently describing symptoms as insomnia and being more likely to receive treatment for depression rather than OSA [27]. Our finding of no significant differences between sexes underscored the importance for increased attention to OSA in female patients. However, we recognize this as an important area for future research with larger cohorts.

Twenty-five percents of participants in our study had their REI increased above 5 events/hour following MS and BS. Some of these individual increases were insignificant and could be a result of nightly variation that happened to cross the OSA diagnostic threshold of 5 events/hour. Lechat et al. raised concerns regarding night-to-night variability in the diagnosis of OSA and suggested that a single-night sleep test could lead to misdiagnosis in approximately 20–50% of cases [28]. In this study, we categorized participants into 3 groups based on the degree of changes in REI and 3% ODI after surgery: clinically significant increase (Δ ≥ 2.5 events/hour), clinically significant decrease (Δ ≤ -2.5 events/hour) and clinically insignificant change (-2.5 < Δ < 2.5 events/hour) and compared the mean surgical movement of the jaws and other pre-operative parameters in each group. These analyses were performed to discern if any of these variables could be used to predict such REI and 3% ODI changes. Our result showed that participants with a clinically significant reduction in REI (ΔREI ≤ -2.5 events/hour) exhibited significantly higher pre-surgical REI compared to those with minor REI changes (-2.5 < ΔREI < 2.5 events/hour). This finding is consistent with a previous study suggesting that individuals with initially higher AHI tended to experience greater reduction in AHI post-operatively [29]. Additionally, the night-to-night variability may also contribute to the observed greater reduction. Tschopp and coworkers demonstrated that the AHI could vary as much as 57% across different nights [30]. No other variable differences were observed among the 3 groups with different degrees of REI changes. This study also demonstrated no significant difference in surgical distance, pre-operative sleep-related respiratory parameters and body metrics, among the 3 groups of participants categorized by the degree of changes in 3% ODI. These results suggested the impact of surgical correction for skeletal class III malocclusion on 3% ODI was potentially within the threshold of physiological adaptation, given that our subjects consisted of young and non-obese individuals with adaptive potential. Additionally, the limited sample size could have influenced the ability to detect the statistically significant difference.

The result of the non-significant change in sleep-related respiratory parameters after the surgery has been discussed as possibly attributable to an initial significantly larger airway in patients with skeletal class III and post-operative biological adaptations. The observed post-operative inferoposterior hyoid bone displacement and associated protrusive head position could serve as compensatory mechanism that preserved pharyngeal airway dimensions and reduced susceptibility to collapse [13, 15, 18]. However, physiological adaptation varies among individuals [18]. The findings of this study may not generalize to obese and older subjects and those with underlying medical conditions or craniofacial anomalies since our samples included mostly young adults with normal BMI. A recent study showed that sleep duration and deep sleep stages tended to decrease with age, and potentially increased the likelihood of developing sleep disorders [31]. Non-anatomical factors such as impaired pharyngeal muscle responsiveness, unstable ventilatory control, and low arousal threshold could also explain the variability of outcomes among different individuals [32].

We employed HST (Nox-T3) to evaluate sleep-related respiratory parameters in this study due to convenience and ability to recruit more participants. However, sleep-related respiratory events examined using total analyzed time, and hypopneas identified without cortical arousal detection, could lead to an underestimation of REI compared to AHI obtained from type I PSG [5]. Our study investigated the impact of skeletal class III surgical correction on PSG parameters rather than changes observed in radiographic imaging such as 2D cephalogram or 3D cone-beam computed tomography. Radiographic examinations capture patients in an awake and upright position which do not accurately reflect neuromuscular tone, airway collapsibility, or dynamic airway function during sleep. Additional strength of this study included a prospective design with sample size calculation to ensure adequate power to detect meaningful differences. The prospective cohort design allows investigations to be conducted in a real-world setting with less cost and fewer resources. It also has better control of confounding factors. Therefore, this research design enhances the validity of the conclusions drawn from the study.

The amounts of jaw movement in this study were obtained from virtual surgical plans. However, past study demonstrated no statistically significant difference between the virtual surgical plan and the post-operative result [33]. Further studies should focus on other racial and ethnic groups, and individuals with more advanced age, and higher BMI. Type I sleep study should also be considered to increase the accuracy of PSG parameters. Finally, longer-term studies with extended follow-up periods are warranted.

## Conclusion

This study provided evidence that mandibular setback surgery, with or without maxillary advancement, did not have a significant impact on sleep-related respiratory parameters in young and non-obese individuals. Correlations were observed between pre-operative ESS and changes in REI, average SpO_2_, and snoring percentage. Additionally, no significant sex difference was detected regarding changes in the PSG parameters following the surgery. We also found that participants who experienced a significant decrease in REI (ΔREI ≤ -2.5) had significantly higher pre-surgical REI compared to those with minor REI changes.

## Data Availability

The data that supports the findings of this study are available on request from the corresponding author.
